# Association of Silica Dust Exposure and Cigarette Smoking With Mortality Among Mine and Pottery Workers in China

**DOI:** 10.1001/jamanetworkopen.2020.2787

**Published:** 2020-04-14

**Authors:** Dongming Wang, Meng Yang, Yuewei Liu, Jixuan Ma, Tingming Shi, Weihong Chen

**Affiliations:** 1 Department of Occupational and Environmental Health, School of Public Health, Tongji Medical College, Huazhong University of Science and Technology, Wuhan, Hubei, China; 2 Key Laboratory of Environment and Health, Ministry of Education and Ministry of Environmental Protection, State Key Laboratory of Environmental Health, School of Public Health, Tongji Medical College, Huazhong University of Science and Technology, Wuhan, Hubei, China; 3 Department of Epidemiology, School of Public Health, Sun Yat-sen University, Guangzhou, China; 4 Institute of Health Surveillance, Analysis and Protection, Hubei Center for Disease Control and Prevention, Wuhan, Hubei, China

## Abstract

**Importance:**

The joint association of long-term silica dust exposure and cigarette smoking with mortality has not been well established.

**Objective:**

To evaluate the joint association of silica dust exposure and cigarette smoking with mortality in a large cohort of workers at mines and factories in China.

**Design, Setting, and Participants:**

This cohort study included 44 708 adults who were employed in 20 metal mines and 9 pottery factories in central and southern China for at least 1 year between January 1, 1960, and December 31, 1974. Participants were retrospectively followed up to January 1, 1960, and prospectively followed up to December 31, 2003. Data analysis was conducted from April 5, 2019, to October 26, 2019.

**Exposures:**

Cumulative respirable silica dust exposure was estimated by linking a job-exposure matrix to participants’ personal work histories. Cigarette smoking data were collected through participant questionnaires.

**Main Outcomes and Measures:**

The main outcome was mortality, with codes from the *International Classification of Diseases, Tenth Revision, Clinical Modification* (*ICD-10-CM*) used to categorize diseases associated with mortality.

**Results:**

Among 44 708 participants, 38 221 (85.49%) were men, with a mean (SD) age at cohort entrance of 26.9 (8.1) years. A total of 13 700 deaths were observed during 1 534 005 person-years of follow-up, with a median follow-up period of 34.9 years (range, 4.8-43.9 years). Silica exposure was associated with a higher risk of mortality among individuals with all diseases, lung cancer, respiratory tuberculosis, cardiovascular diseases, and diseases of the respiratory system; cigarette smoking was associated with an increased risk of mortality among individuals with all diseases, lung cancer, respiratory tuberculosis, cerebrovascular diseases, and diseases of the respiratory tract. The hazard ratios for the joint association of silica dust exposure and cigarette smoking with mortality were 4.51 (95% CI, 3.23-6.29) for lung cancer, 3.21 (95% CI, 2.53-4.08) for certain infectious and parasitic diseases, 3.93 (95% CI, 2.99-5.15) for respiratory tuberculosis, 6.27 (95% CI, 4.83-8.15) for diseases of the respiratory system, and 12.52 (95% CI, 7.92-19.80) for pneumoconiosis, with a significant additive interaction (*P* < .001). The proportions of the joint association for the additive interaction of silica dust exposure and cigarette smoking were 21.63% for lung cancer, 42.12% for certain infectious and parasitic diseases, 42.25% for respiratory tuberculosis, 29.55% for diseases of the respiratory system, and 36.46% for pneumoconiosis.

**Conclusions and Relevance:**

These findings suggest that cigarette smoking is associated with an increased risk of mortality in individuals exposed to silica dust. Smoking cessation and the control of silica dust concentrations may be important for reducing the risk of mortality among individuals exposed to silica.

## Key Points

**Question:**

Is there an association of silica dust exposure and cigarette smoking with mortality?

**Findings:**

In this cohort study of 44 708 adults who worked in mines or pottery factories in China and were followed up for a median of 34.9 years, the combination of silica dust exposure and cigarette smoking was found to be associated with mortality among individuals with lung cancer, certain infectious and parasitic diseases, respiratory tuberculosis, diseases of the respiratory system, and pneumoconiosis.

**Meaning:**

Smoking cessation and control of silica dust concentrations may be associated with reduced risk of mortality among individuals exposed to silica dust.

## Teaser

This cohort study examines the joint association of silica dust exposure and cigarette smoking with mortality among a cohort of adults who worked in mines or pottery factories in China between 1960 and 1974, with a total follow-up period of 44 years.

## Introduction

Crystalline silica is one of the most common minerals on earth, and exposure to this mineral widely occurs among individuals in a variety of industrial occupations, such as mining, pottery making, sandblasting, rock drilling, tunneling operations, construction, and metal casting.^1^ Exposure to crystalline silica dust is regarded as one of the most serious occupational hazards in the workplace.^2^ The US Occupational Safety and Health Administration estimated that 2.3 million US individuals had been exposed to silica dust as of 2016.^3^ In China, it was estimated that more than 23 million individuals had been exposed directly and indirectly to silica dust in the workplace as of 2009.^4^ The adverse health effects associated with silica exposure have been an increasing public health concern for decades. Long-term exposure to silica dust has been associated with a higher risk of mortality among patients with silicosis,^5^ pulmonary tuberculosis,^6^ cardiovascular disease,^7^ and lung cancer.^8^

Cigarette smoking is also a public health concern around the world. According to the World Health Organization, approximately 320 million people in China smoke cigarettes, which represents one-third of the total number of individuals who smoke worldwide.^9^ Data have indicated that cigarette smoking is also associated with mortality in patients with lung cancer,^10^ chronic obstructive pulmonary disease,^11^ and cardiovascular disease.^12^ Moreover, published studies have reported that the rate of smoking among individuals employed in industrial settings is high compared with that of the general population.^13^ A previous study indicated that approximately 62% of individuals who worked in metal mines and pottery factories had a history of smoking.^14^ However, the joint association of silica exposure and cigarette smoking with mortality has not been well assessed to date. Therefore, we conducted a large cohort study of adults who worked in metal mines and pottery factories to evaluate the independent and joint associations of silica dust exposure and cigarette smoking with mortality.

## Methods

### Study Population

This study was part of a larger cohort study performed in China; results of the larger study have been reported elsewhere.^14,15^ In brief, the cohort was established in 1986, and it included 74 040 individuals who worked in 20 metal mines and 9 pottery factories in central and southern China for 1 year or more between January 1, 1960, and December 31, 1974. The cohort was retrospectively followed up to January 1, 1960, and prospectively followed up to December 31, 2003. In the present study, we excluded 8268 individuals without detailed work histories and 21 063 individuals without detailed information about smoking history, resulting in a final sample of 44 708 individuals. The study was approved by the institutional review boards of Tongji Medical College and the US Occupational Safety and Health Administration, with written informed consent obtained before participants were interviewed. This study followed the Strengthening the Reporting of Observational Studies in Epidemiology (STROBE) reporting guideline.

### Silica Exposure

The methods used to assess silica exposure were described in detail in a previously published study.^16^ In brief, data abstraction forms were developed to collect information about historical and current silica exposure and employees' work histories beginning in 1950. A job-exposure matrix was then constructed using facility, job title, and calendar year combinations. Silica exposure was estimated using information about the concentration of silica content in total dust among individuals with similar job titles or, if data regarding the year or job title were missing (<20% of individuals), among individuals with the same job title in different years.

Work histories for each individual were obtained from employment records in the mine and factory files. Individual histories included job titles and duration of full employment. After linking this information with the job-exposure matrix, the estimated cumulative respirable silica dust exposure (cumulative dust exposure), measured in milligrams per cubic meter per year, was calculated for each individual as follows:
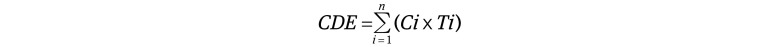
in which *CDE* represents cumulative dust exposure, *n* represents the total number of lifetime job titles, *Ci* represents the 8-hour time-weighted mean concentration of silica dust associated with the *i*th job title within a facility and employment period, and *Ti* represents the duration of working years for the *i*th job title.

We calculated cumulative dust exposure from the start date of silica-exposed employment to the end date of employment, the date of loss to follow-up, the date of death, or the end of the follow-up period (December 31, 2003).

### Cigarette Smoking

The detailed lifetime smoking data were collected in 1986, 1995, and 2004.^17^ The smoking data from 11% of individuals who had died were obtained from their colleagues or next of kin. Data reliability was examined in 2004 using information from 1990 randomly selected participants. The percentage of agreement on smoking status (yes or no) was 93.6% for living individuals (based on self-reported data) and 89.1% for deceased individuals (based on data obtained from the individual’s next of kin or colleagues).

Individuals who had ever smoked were defined as those who had at any point in their lifetimes smoked cigarettes regularly for 1 year, including current and former smokers. Individuals who currently smoked were defined as those who had not stopped smoking or had stopped smoking within 1 year before the end of follow-up.

### Mortality

The vital status of all individuals was tracked by local health care practitioners during the follow-up period. Information about mortality was collected from hospital medical records (60.5%); employment registers, accident records, or death certificates (35.2%); and oral reports from participants’ relatives (4.3%).^14^ *International Classification of Diseases, Tenth Revision, Clinical Modification* (*ICD-10-CM*) codes were used to categorize causes of death.

### Statistical Analysis

The sociodemographic characteristics of participants were reported as mean (SD) for continuous variables and number (percentage) for categorical variables. Exposure-response analyses were conducted for silica dust exposure and risk of mortality, and age was used as the time variable to define the risk sets for mortality.^7,18^ Cox proportional hazards models were used to calculate hazard ratios (HRs) and 95% CIs for cumulative dust exposure (time-dependent) and the risk of selected causes of death, with adjustment for sex, year of hire (1950 or earlier, 1951-1960, 1961-1970, and 1971 or later), age at hire (continuous), type of facility (tungsten mine, iron and/or copper mine, tin mine, and pottery factory), and smoking history (pack-years). Cumulative dust exposure was categorized into 4 groups based on the percentiles from the exposure distribution (the final cumulative dust exposure). The linear trend tests were conducted by including the median value for each level of dust exposure as a continuous variable in the models. In addition, we assessed the nonlinear association between cumulative dust exposure and mortality by using restricted cubic splines with 4 knots at the 5th, 35th, 65th, and 95th percentiles of the distribution.^19^

The risk of mortality among individuals who had ever smoked was estimated in comparison with those who had never smoked. The HRs and 95% CIs were calculated by adjusting for sex, year of hire (1950 or earlier, 1951-1960, 1961-1970, and 1971 or later), age at hire (continuous), type of facility (tungsten mine, iron and/or copper mine, tin mine, and pottery factory), and cumulative dust exposure (continuous).

To investigate the joint association of silica exposure and cigarette smoking, we estimated HRs by crossed dichotomized silica exposure (individuals who were exposed vs individuals who were not exposed) and smoking status (individuals who had ever smoked vs individuals who had never smoked). The relative excess risk due to interaction (RERI) was used to assess the additive interaction,^20^ and we considered silica dust exposure and cigarette smoking as 2 continuous variables. We also examined the decomposition of the joint association by evaluating the proportion associated with silica dust exposure alone, cigarette smoking alone, and the interaction of silica dust exposure and cigarette smoking.^20^ In brief, the HRs for silica exposure, cigarette smoking, and their combination were assigned numbers, with HR_01_ representing silica dust exposure alone, HR_10_ representing cigarette smoking alone, and HR_11_ representing the combination of silica dust exposure and cigarette smoking. The joint excess relative risk for both silica dust exposure and cigarette smoking (HR_11_ − 1) was decomposed into the excess relative risk for silica dust exposure alone (HR_01_ − 1), cigarette smoking alone (HR_10_ − 1), and RERI ([HR_11_ − HR_01_ − HR_10_] + 1). The proportions of the joint association due to silica dust exposure alone ([HR_01_ – 1]/[HR_11_ – 1]), cigarette smoking alone ([HR_10_ – 1]/[HR_11_ – 1]), and their additive interaction (RERI/[HR_11_ − 1]) were calculated. All tests were 2-sided and paired, with a significance threshold of *P* < .05. All statistical analyses were performed using SAS statistical software, version 9.4 (SAS Institute). Data analysis was conducted from April 5, 2019, to October 26, 2019.

## Results

A total of 44 708 participants (mean [SD] age at cohort entrance, 26.9 [8.1] years; 38 221 men [85.49%]) were included in this study. Demographic characteristics of participants are summarized in Table 1. A total of 3172 individuals (7.09%) were still employed at the end of follow-up. Among all participants, 29 182 individuals (65.27%) were exposed to silica dust during the production process, and the mean (SD) cumulative dust exposure was 3.64 (4.17) mg/m^3^ per year. The percentage of individuals who had ever smoked was 61.68%, with a mean (SD) of 32.7 (16.6) pack-years. A total of 13 700 deaths were observed during 1 534 005 person-years of follow-up, with a median follow-up period of 34.9 years (range, 4.8-43.9 years). Among all participants, the mortality rate was 893 per 100 000 person-years, with a rate of 1042 deaths per 100 000 person-years among individuals who were exposed to silica compared with 609 deaths per 100 000 person-years among individuals who were not exposed. The mortality rate was 1034 deaths per 100 000 person-years among individuals who had ever smoked compared with 668 deaths per 100 000 person-years among individuals who had never smoked.

**Table 1.** Participant Characteristics^a^

Characteristic	Participants				
	Total (N = 44 708)	Level of CDE^b^			
		Unexposed (n = 15 526)	Low (n = 9727)	Medium (n = 9728)	High (n = 9727)
Male	38 221 (85.49)	11 115 (71.59)	9101 (93.56)	9104 (93.59)	8901 (91.51)
Age, mean (SD)					
At hire	24.5 (7.3)	23.6 (7.1)	23.4 (6.0)	24.5 (6.9)	26.8 (8.5)
At first silica exposure^c^	22.9 (6.9)	NA	24.6 (7.3)	23.2 (6.4)	20.9 (6.5)
At first diagnosis of pneumoconiosis^d^	45.5 (10.3)	NA	48.9 (9.1)	47.1 (10.1)	44.6 (10.3)
At last silica exposure^c^	43.0 (10.6)	NA	40.4 (11.1)	43.8 (10.1)	44.7 (10.1)
Pneumoconiosis diagnosis^d^	6477 (14.49)	NA	377 (3.87)	1723 (17.71)	4377 (45.00)
Year of birth, median (range)	1937 (1900-1963)	1940 (1900-1960)	1943 (1900-1963)	1936 (1900-1959)	1930 (1900-1959)
Year of hire					
1915-1950	1004 (2.25)	205 (1.32)	32 (0.33)	120 (1.23)	647 (6.65)
1951-1960	22 782 (50.96)	6653 (42.85)	3153 (32.41)	5795 (59.57)	7181 (73.83)
1961-1970	11 192 (25.03)	4436 (28.57)	3137 (32.25)	2133 (21.93)	1486 (15.28)
1971-1975	9730 (21.76)	4232 (27.26)	3405 (35.01)	1680 (17.27)	413 (4.25)
Median (range)	1958 (1916-1975)	1962 (1916-1975)	1967 (1932-1975)	1958 (1933-1975)	1955 (1923-1975)
Status at end of follow-up					
Employed	3172 (7.09)	1725 (11.11)	726 (7.46)	566 (5.82)	155 (1.59)
Unemployed	4991 (11.16)	3485 (22.45)	877 (9.02)	445 (4.57)	184 (1.89)
Retired	22 845 (51.1)	7113 (45.81)	6033 (62.02)	5521 (56.75)	4178 (42.95)
Died	13 700 (30.64)	3203 (20.63)	2091 (21.50)	3196 (32.85)	5210 (53.56)
Year of first silica exposure, median (range)^c^	1957 (1915-2000)	NA	1968 (1923-2000)	1957 (1919-1986)	1951 (1915-1983)
Duration of silica exposure, mean (SD), y^c^	18.9 (10.3)	NA	14.8 (9.9)	19.2 (9.5)	22.8 (9.9)
Cumulative total silica exposure, mean (SD), mg/m^3^ per y^c^	147.8 (166.7)	NA	44.8 (41.8)	85.8 (53.8)	312.8 (192.5)
CDE, mean (SD), mg/m^3^ per y^c^	3.6 (4.2)	NA	0.6 (0.3)	2.1 (0.8)	8.3 (4.2)
Smoking status					
Ever smoked	27 578 (61.68)	7338 (47.26)	6505 (66.88)	6867 (70.59)	6868 (70.61)
Never smoked	17 130 (38.32)	8188 (52.74)	3222 (33.12)	2861 (29.41)	2859 (29.39)
Duration of smoking, mean (SD), pack-years	32.7 (16.6)	32.7 (17.2)	31.9 (16.4)	34.3 (16.9)	31.8 (15.6)

Abbreviation: CDE, cumulative respirable silica dust exposure; NA, not applicable.

^a^ Data are presented as number (percentage) or participants unless otherwise indicated.

^b^ The level of CDE comprised the tertiles of exposure among all individuals exposed to silica dust. Low exposure was defined as 0 to 1.056 mg/m^3^ per year, medium exposure as 1.057-3.925 mg/m^3^ per year, and high exposure as more than 3.925 mg/m^3^ per year.

^c^ Results among individuals exposed to silica dust only.

^d^ Results among individuals with pneumoconiosis only.

Table 2 shows the HRs and 95% CIs for mortality associated with cumulative dust exposure. The primary diseases associated with mortality were malignant neoplasms, cardiovascular diseases, diseases of the respiratory system, cerebrovascular diseases, and certain infectious and parasitic diseases. Positive associations between exposure and response were found between silica dust exposure and several diseases. Compared with participants who were not exposed to silica dust, the risk of mortality among individuals exposed to silica dust was significantly higher except among those with certain infectious and parasitic diseases and low exposure and those with cardiovascular diseases and low exposure: all diseases (low exposure: HR, 1.14 [95% CI, 1.08-1.21]; medium exposure: HR, 1.20 [95% CI, 1.13-1.26]; and high exposure: HR, 1.54 [95% CI, 1.47-1.62]), lung cancer (low exposure: HR, 1.32 [95% CI, 1.07-1.62]; medium exposure: HR, 1.51 [95% CI, 1.25-1.83]; and high exposure: HR, 1.52 [95% CI, 1.24-1.87]), certain infectious and parasitic diseases (low exposure: HR, 1.18 [95% CI, 0.94-1.46]; medium exposure: HR, 1.90 [95% CI, 1.60-2.26]; and high exposure: HR, 3.66 [95% CI, 3.13-4.28]), cardiovascular diseases (low exposure: HR, 1.03 [95% CI, 0.89-1.18]; medium exposure: HR, 1.21 [95% CI, 1.07-1.36]; and high exposure: HR, 1.84 [95% CI, 1.66-2.05]), and diseases of the respiratory system (low exposure: HR, 1.58 [95% CI, 1.30-1.93]; medium exposure: HR, 3.17 [95% CI, 2.69-3.73]; and high exposure: HR, 6.41 [95% CI, 5.49-7.51]). In addition, the nonlinear association between cumulative dust exposure and the risk of mortality was assessed by a spline curve, which indicated that the risk of mortality associated with all diseases, respiratory tuberculosis, cardiovascular diseases (including pulmonary heart disease), and pneumoconiosis increased as the cumulative dust exposure increased (Figure).

**Table 2.** Association of Cumulative Respirable Silica Dust Exposure With Mortality^**a**^

Disease (*ICD-10-CM* code)	Deaths, No.	Level of CDE vs nonexposure, HR (95% CI)^b^			P value
		Low	Medium	High	
Malignant neoplasm (C00-C97)	2949	1.21 (1.09-1.35)	1.20 (1.08-1.33)	1.01 (0.90-1.13)	.27
Malignant neoplasm of nasopharynx (C11)	139	0.85 (0.52-1.37)	0.70 (0.44-1.10)	0.62 (0.37-1.05)	.12
Malignant neoplasm of liver and intrahepatic bile ducts (C22)	766	1.18 (0.96-1.46)	1.16 (0.95-1.42)	0.85 (0.68-1.07)	.04
Lung cancer (C33-C34)	917	1.32 (1.07-1.62)	1.51 (1.25-1.83)	1.52 (1.24-1.87)	<.001
Certain infectious and parasitic diseases (A00-B99, J65)	2045	1.18 (0.94-1.46)	1.90 (1.60-2.26)	3.66 (3.13-4.28)	<.001
Respiratory tuberculosis (A15-A16, J65)	1857	1.14 (0.88-1.47)	2.13 (1.76-2.58)	4.30 (3.62-5.11)	<.001
Cardiovascular disease (I00-I52, I70-I99)	2948	1.03 (0.89-1.18)	1.21 (1.07-1.36)	1.84 (1.66-2.05)	<.001
Pulmonary heart disease (I26-I27)	1585	1.20 (0.92-1.57)	1.87 (1.53-2.29)	4.16 (3.48-4.97)	<.001
Hypertensive heart disease (I11)	329	0.78 (0.52-1.16)	0.83 (0.61-1.14)	0.86 (0.65-1.14)	.56
Ischemic heart disease (I20-I25)	516	1.20 (0.94-1.55)	1.21 (0.95-1.54)	0.77 (0.58-1.01)	.008
Chronic rheumatic heart disease (I05-I09)	92	0.83 (0.40-1.71)	1.20 (0.69-2.07)	0.71 (0.39-1.30)	.21
Cerebrovascular disease (I60-I69)	2160	1.01 (0.88-1.15)	0.94 (0.83-1.06)	0.90 (0.80-1.02)	.09
Respiratory system disease (J00-J99)	2727	1.58 (1.30-1.93)	3.17 (2.69-3.73)	6.41 (5.49-7.51)	<.001
Pneumoconiosis (J60-J65)	1601	[Reference]	5.08 (3.60-7.13)	15.25 (10.72-21.49)	<.001
Digestive system disease (K00-K93)	641	1.21 (0.96-1.53)	0.94 (0.74-1.18)	0.88 (0.70-1.11)	.12
External cause (V01-Y98)	797	1.55 (1.28-1.87)	1.32 (1.07-1.63)	1.03 (0.81-1.31)	.29
All diseases (A00-Y98)	13 700	1.14 (1.08-1.21)	1.20 (1.13-1.26)	1.54 (1.47-1.62)	<.001

Abbreviations: CDE, cumulative respirable silica dust exposure; HR, hazard ratio; *ICD-10-CM*, *International Classification of Diseases, Tenth Revision, Clinical Modification*.

^a^ Adjusted for sex, year at hire (1950 or earlier, 1951-1960, 1961-1970, and 1971 or later), age at hire (continuous), type of facility (tungsten mine, iron and/or copper mine, tin mine, and pottery factory), and smoking intensity (continuous).

^b^ The level of CDE comprised the tertiles of exposure among all individuals exposed to silica dust. Low exposure was defined as 0 to 1.056 mg/m^3^ per year, medium exposure as 1.057-3.925 mg/m^3^ per year, and high exposure as more than 3.925 mg/m^3^ per year.

**Figure.**
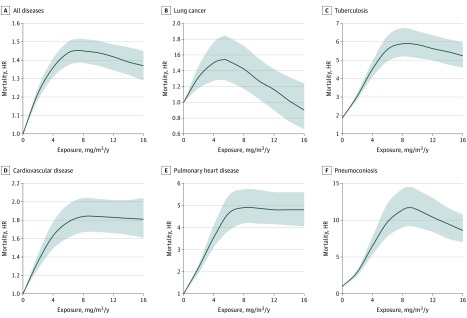
Association Between Cumulative Respirable Silica Dust Exposure and Mortality Multivariable spline curves were adjusted for sex, year of hire (1950 or earlier, 1951-1960, 1961-1970, and 1971 or later), age at hire (continuous), type of facility (tungsten mine, iron and/or copper mine, tin mine, and pottery factory), and length of smoking (pack-years). In all graphs, solid curves indicate hazard ratios (HRs) and shaded areas indicate 95% CIs.

Table 3 shows the HRs and 95% CIs for mortality associated with cigarette smoking. Compared with individuals who had never smoked, the risk of mortality among individuals who had ever smoked was significantly higher for all diseases (HR, 1.26; 95% CI, 1.21-1.31), malignant neoplasms (HR, 1.71; 95% CI, 1.56-1.87), certain infectious and parasitic diseases (HR, 1.56; 95% CI, 1.40-1.74), cerebrovascular diseases (HR, 1.35; 95% CI, 1.22-1.49), and diseases of the respiratory system (HR, 1.68; 95% CI, 1.53-1.85) after adjusting for potential confounders. In addition, the association with mortality was stronger among individuals with lung cancer (HR, 3.27; 95% CI, 2.66-4.01), respiratory tuberculosis (HR, 1.65; 95% CI, 1.47-1.85), and pneumoconiosis (HR, 1.81; 95% CI, 1.59-2.05).

**Table 3.** Association of Cigarette Smoking With Mortality^**a**^

Disease (*ICD-10-CM* code)	Deaths, No. (%)	Hazard ratio (95% CI)^b^
Malignant neoplasm (C00-C97)	2248 (8.15)	1.71 (1.56-1.87)
Malignant neoplasm of nasopharynx (C11)	103 (0.37)	1.35 (0.90-2.02)
Malignant neoplasm of liver and intrahepatic bile ducts (C22)	564 (2.05)	1.34 (1.13-1.60)
Lung cancer (C33-C34)	788 (2.86)	3.27 (2.66-4.01)
Certain infectious and parasitic diseases (A00-B99, J65)	1588 (5.76)	1.56 (1.40-1.74)
Respiratory tuberculosis (A15-A16, J65)	1459 (5.29)	1.65 (1.47-1.85)
Cardiovascular disease (I00-I52, I70-I99)	1975 (7.16)	1.04 (0.96-1.13)
Pulmonary heart disease (I26-I27)	1100 (3.99)	1.07 (0.96-1.20)
Hypertensive heart disease (I11)	198 (0.72)	0.84 (0.66-1.07)
Ischemic heart disease (I20-I25)	337 (1.22)	1.01 (0.83-1.23)
Chronic rheumatic heart disease (I05-I09)	51 (0.18)	0.85 (0.53-1.37)
Cerebrovascular disease (I60-I69)	1517 (5.50)	1.35 (1.22-1.49)
Respiratory system disease (J00-J99)	2152 (7.80)	1.68 (1.53-1.85)
Pneumoconiosis (J60-J65)	1308 (4.74)	1.81 (1.59-2.05)
Digestive system disease (K00-K93)	443 (1.61)	1.06 (0.89-1.27)
External causes (V01-Y98)	493 (1.79)	0.75 (0.65-0.87)
All diseases (A00-Y98)	9763 (35.40)	1.26 (1.21-1.31)

Abbreviation: *ICD-10-CM*, *International Classification of Diseases, Tenth Revision, Clinical Modification*.

^a^ Adjusted for sex, year at hire (1950 or earlier, 1951-1960, 1961-1970, and 1971 or later), age at hire (continuous), type of facility (tungsten mine, iron and/or copper mine, tin mine, and pottery factory), and silica dust (continuous).

^b^ Hazard ratios were calculated for individuals who had ever smoked vs individuals who had never smoked.

The joint association of silica dust exposure and smoking with mortality is shown in Table 4. Compared with individuals who were not exposed to silica and had never smoked, the risk of mortality among individuals who were exposed to silica and had ever smoked was significantly higher for all diseases (HR, 1.58; 95% CI, 1.48-1.69), malignant neoplasms (HR, 1.87; 95% CI, 1.62-2.16), certain infectious and parasitic diseases (HR, 3.21; 95% CI, 2.53-4.08), and diseases of the respiratory system (HR, 6.27; 95% CI, 4.83-8.15) and especially for lung cancer (HR, 4.51; 95% CI, 3.23-6.29), respiratory tuberculosis (HR, 3.93; 95% CI, 2.99-5.15), and pneumoconiosis (HR, 12.52; 95% CI, 7.92-19.80). With regard to the additive interaction, the RERI of silica dust exposure and cigarette smoking was 0.76 (95% CI, 0.22-1.49) for lung cancer, 0.93 (95% CI, 0.25-1.67) for certain infectious and parasitic diseases, 1.24 (95% CI, 0.27-2.32) for respiratory tuberculosis, 1.56 (95% CI, 0.62-2.32) for diseases of the respiratory system, and 4.20 (95% CI, 3.02-5.48) for pneumoconiosis, with only the association for pneumoconiosis being statistically significant.

**Table 4.** Joint Association of Cumulative Respirable Silica Dust Exposure and Cigarette Smoking With Mortality

Disease (*ICD-10-CM* code)	Smoking status, HR (95% CI)		Additive interaction, RERI (95% CI)
	Never smoked	Ever smoked	
Malignant neoplasm (C00-C97)			
Unexposed	[Reference]	1.69 (1.45 to 1.98)	0.08 (−0.44 to 0.63)
Exposed	1.10 (0.94 to 1.29)	1.87 (1.62 to 2.16)	
Lung cancer (C33-C34)			
Unexposed	[Reference]	3.32 (2.34 to 4.71)	0.76 (0.22 to 1.49)
Exposed	1.43 (0.99 to 2.08)	4.51 (3.23 to 6.29)	
Certain infectious and parasitic diseases (A00-B99, J65)			
Unexposed	[Reference]	1.21 (0.91 to 1.62)	0.93 (0.25 to 1.67)
Exposed	2.07 (1.61 to 2.65)	3.21 (2.53 to 4.08)	
Respiratory tuberculosis (A15-A16, J65)			
Unexposed	[Reference]	1.27 (0.91 to 1.76)	1.24 (0.27 to 2.32)
Exposed	2.42 (1.82 to 3.20)	3.93 (2.99 to 5.15)	
Cardiovascular disease (I00-I52, I70-I99)			
Unexposed	[Reference]	1.16 (0.98 to 1.38)	−0.18 (−0.91 to 0.64)
Exposed	1.56 (1.35 to 1.81)	1.54 (1.33 to 1.78)	
Pulmonary heart disease (I26-I27)			
Unexposed	[Reference]	1.47 (1.05 to 2.05)	−0.54 (−1.38 to 0.37)
Exposed	3.62 (2.71 to 4.82)	3.54 (2.67 to 4.70)	
Ischemic heart disease (I20-I25)			
Unexposed	[Reference]	1.12 (0.80 to 1.57)	−0.17 (−0.95 to 0.81)
Exposed	1.16 (0.84 to 1.60)	1.11 (0.82 to 1.51)	
Cerebrovascular disease (I60-I69)			
Unexposed	[Reference]	1.45 (1.22 to 1.71)	−0.14 (−0.91 to 0.87)
Exposed	0.98 (0.83 to 1.16)	1.29 (1.11 to 1.51)	
Respiratory system disease (J00-J99)			
Unexposed	[Reference]	1.75 (1.29 to 2.36)	1.56 (0.62 to 2.32)
Exposed	3.97 (3.03 to 5.19)	6.27 (4.83 to 8.15)	
Pneumoconiosis (J60-J65)^a^			
Unexposed	[Reference]	1.77 (1.09 to 2.87)	4.20 (3.02 to 5.48)
Exposed	7.56 (4.73 to 12.08)	12.52 (7.92 to 19.80)	
All diseases (A00-Y98)			
Unexposed	[Reference]	1.24 (1.15 to 1.34)	0.07 (−0.54 to 0.81)
Exposed	1.27 (1.18 to 1.36)	1.58 (1.48 to 1.69)	

Abbreviations: HR, hazard ratio; *ICD-10-CM*, *International Classification of Diseases, Tenth Revision, Clinical Modification*; RERI, relative excess risk due to interaction.

^a^ For pneumoconiosis, the dichotomous analysis was conducted on the basis of silica exposure (lower or higher level divided by 1.895 mg/m^3^ per year) and smoking status (ever smoked vs never smoked), and the reference category was defined as the lower silica exposure and nonsmoking levels.

For individuals with lung cancer, the proportions of the joint association were 12.29% for silica dust exposure alone, 66.08% for cigarette smoking alone, and 21.63% for their interaction. For individuals with certain infectious and parasitic diseases, the proportions were 48.26% for silica exposure alone, 9.62% for cigarette smoking alone, and 42.12% for their interaction. For individuals with respiratory tuberculosis, the proportions were 48.43% for silica exposure alone, 9.12% for cigarette smoking alone, and 42.45% for their interaction. For individuals with diseases of the respiratory system, the proportions were 56.28% for silica exposure alone, 14.17% for cigarette smoking alone, and 29.55% for their interaction. For individuals with pneumoconiosis, the proportions were 56.89% for silica dust exposure alone, 6.65% for cigarette smoking alone, and 36.46% for their interaction.

## Discussion

In the present cohort study with a large sample and a long-term follow-up period, we identified a positive association between cumulative silica dust exposure and the risk of mortality among individuals with all diseases, lung cancer, certain infectious and parasitic diseases, cardiovascular diseases, and diseases of the respiratory system. Among individuals who had ever smoked, a higher risk of mortality was also found among those with all diseases, lung cancer, certain infectious and parasitic diseases, cerebrovascular diseases, and diseases of the respiratory system. Furthermore, our results indicated that the additive interaction of silica exposure and cigarette smoking was associated with a higher risk of mortality among those with lung cancer, certain infectious and parasitic diseases, respiratory tuberculosis, diseases of the respiratory system, and pneumoconiosis.

The association between cumulative dust exposure and the risk of mortality for all diseases, lung cancer, certain infectious and parasitic diseases, cardiovascular diseases, and diseases of the respiratory system has been reported in a previous study conducted among 74 040 individuals.^14^ The present study supported those results when the analyses were further adjusted for cigarette smoking, which suggests that our results are reliable and stable. The findings of our study may provide useful information for the prevention and control of silica exposure.

It has been reported that cigarette smoking is associated with adverse health effects for multiple organs and systems.^21^ According to the Centers for Disease Control and Prevention,^22^ cigarette smoking is associated with more than 480 000 deaths each year in the US. Smoking has also been reported to be associated with approximately 1 million deaths in China each year,^23^ which represents approximately 1 in 5 deaths worldwide. In addition, cigarette smoking has been associated with mortality in individuals with diseases of the respiratory system, including chronic obstructive pulmonary disease and lung cancer, because smoking damages the airways and the small air sacs.^24,25^ Such associations are also supported by the results of the present study. With regard to respiratory tuberculosis, our findings are consistent with those of other studies that have indicated that smoking may be associated with decreases in the disease immunity of adults and with increases in tuberculosis.^26,27^ In addition, we found that smoking was associated with an increased risk of mortality among individuals with cerebrovascular disease, which was also consistent with the results of other studies.^28,29^ Studies have reported that smoking can damage blood vessels by causing them to thicken and narrow, which may lead to stroke^30^ and coronary heart disease.^31^ However, we did not find a significant association between smoking and the risk of mortality among patients with cardiovascular disease. It is possible that the increased risk of mortality in individuals with respiratory disease and lung cancer partially obscured the risk of mortality in those with cardiovascular disease because we only analyzed the primary disease associated with mortality among participants who had died.

We explored the association of silica dust exposure and cigarette smoking with mortality using additive interaction models. These models indicated that the additive interaction between silica dust exposure and cigarette smoking had a statistically significant association with mortality among patients with lung cancer, certain infectious and parasitic diseases (including respiratory tuberculosis), and diseases of the respiratory system (including pneumoconiosis). We also calculated the proportions of the joint association of these diseases with mortality for silica dust exposure alone, cigarette smoking alone, and their interaction. Of note, the proportions for the joint association with mortality among individuals with pneumoconiosis are not comparable to the proportions for other diseases because the referent group for this mortality category was the lower-exposed group rather than the unexposed group. In a previous study,^32^ the joint association was only evaluated among 7665 individuals who worked in iron mines; the present study may provide more information because it was conducted among a larger sample of individuals who worked in different types of metal mines and pottery factories. In addition, we found a joint association of silica dust exposure and cigarette smoking with mortality among patients with respiratory tuberculosis, which was not found in the previous study.^32^

With regard to individuals with lung cancer, our results were similar to those of the previous study^17^ and other studies^33,34^; however, our study’s focus on mortality among individuals with all diseases may provide more reliable results if a larger sample and longer follow-up period were examined. We believe that the evaluation of the joint association of silica exposure and cigarette smoking with mortality has important implications for public health because smoking is a common public health concern.

### Strengths and Limitations

Our study has several strengths. First, it was a cohort study with a large sample and a long-term follow-up period. Second, detailed information about silica dust exposure and cigarette smoking during the participants’ lifetimes was collected.

This study has limitations. First, long-term exposure to silica dust was evaluated carefully, but measurement errors were inevitable. Silica concentrations before 1950 were estimated by using those reported in 1950, which may have led to the underestimation of silica exposure among the 2% of individuals who worked in mines and pottery factories before 1950. However, the results were almost identical when we excluded individuals whose silica exposure occurred before 1950. Second, because the smoking data for individuals who had died were obtained from their next of kin or colleagues, recall bias might have occurred. Third, the data in our study were analyzed based on the exclusion of 40% of the total population, which may have influenced the results; however, the basic information obtained for individuals in our study and individuals in the total population was comparable. Fourth, we estimated the joint association based on statistical interaction. However, the literature has revealed that interactions estimated as departure from additive models may better reflect biological interactions, and the results may be more accurate.^35,36^ Fifth, the use of personal protective equipment by participants was not considered in our study; however, this equipment was rarely used (<5% of the participants) or improperly used, indicating that the use of personal protective equipment had little influence on the results.

## Conclusions

In the present study, the additive interactions of silica exposure and cigarette smoking were found to be associated with mortality among individuals with lung cancer, certain infectious and parasitic diseases, respiratory tuberculosis, diseases of the respiratory system, and pneumoconiosis. These findings suggest that more effective measures should be taken for the cessation of cigarette smoking and the prevention and control of silica exposure to potentially reduce the risk of mortality.
